# Neuropsychology, social cognition and global functioning among bipolar, schizophrenic patients and healthy controls: preliminary data

**DOI:** 10.3389/fnhum.2013.00661

**Published:** 2013-10-17

**Authors:** Elisabetta Caletti, Riccardo A. Paoli, Alessio Fiorentini, Michela Cigliobianco, Elisa Zugno, Marta Serati, Giulia Orsenigo, Paolo Grillo, Stefano Zago, Alice Caldiroli, Cecilia Prunas, Francesca Giusti, Dario Consonni, A. Carlo Altamura

**Affiliations:** ^1^Department of Neuroscience and Mental Health, Psychiatric Clinic, University of Milan, Fondazione IRCCS Ca' Granda, Ospedale Maggiore PoliclinicoMilan, Italy; ^2^Epidemiology Unit, Department of Preventive Medicine, Fondazione IRCCS Ca' Granda, Ospedale Maggiore PoliclinicoMilan, Italy; ^3^Neurology Unit, Department of Neuroscience and Mental Health, University of Milan, Fondazione IRCCS Ca' Granda, Ospedale Maggiore PoliclinicoMilan, Italy

**Keywords:** schizophrenia, bipolar disorder, social cognition, neuropsychological deficits, ecological tests

## Abstract

This study aimed to determine the extent of impairment in social and non-social cognitive domains in an ecological context comparing bipolar (BD), schizophrenic (SKZ) patients and healthy controls (HC). The sample was enrolled at the Department of Psychiatry of Policlinico Hospital, University of Milan; it includes stabilized SKZ patients (*n* = 30), euthymic bipolar patients (*n* = 18) and HC (*n* = 18). Patients and controls completed psychiatric assessment rating scales, the Brief Assessment of Cognition in Schizophrenia (BACS) and the Executive and Social Cognition Battery (ESCB) that contains both ecological tests of executive function and social cognition, in order to better detect cognitive deficits in patients with normal results in standard executive batteries. The three groups differed significantly for gender and substance abuse, however, the differences did not influence the results. BD patients showed less impairment on cognitive performance compared to SKZ patients, even in “ecological” tests that mimic real life scenarios. In particular, BD performed better than SKZ in verbal memory (*p* < 0.0038) and BACS symbol coding (*p* < 0.0043). Regarding the ESCB tests, in the Hotel task SKZ patients completed significantly less tasks (*p* < 0.001), showed a greater number of errors in Multiple Errands Test (MET-HV) (*p* < 0.0248) and a worse performance in Theory of Mind (ToM) tests (*p* < 0.001 for the Eyes test and Faux pas test). Both patients' groups performed significantly worse than HC. Finally, significant differences were found between the two groups in GAF scores, being greater among BD subjects (*p* < 0.001). GAF was correlated with BACS and ESCB scores showing the crucial role of cognitive and ecological performances in patients' global functioning.

## Introduction

Over the last two decades, there has been an increased interest in neurocognitive functioning and in social cognition (SC) in major psychoses, schizophrenia (SKZ) and bipolar disorder (BD) (Barch and Keefe, 2010; Samamé et al., 2012), diseases causing severe behavioral, relational, and socio-familial disabilities (Altamura et al., 2001). It is widely recognized that SKZ patients exhibit neuropsychological deficits in several cognitive domains, including memory, attention, and executive functions over time (Cornblatt and Keilp, 1994; Addington and Addington, 2000; Kuperberg and Heckers, 2000). Moreover, they experience low levels of performance and a reduced ability to live independently, despite the remission of acute symptomatology, with a negative impact on social and occupational functioning (Heinrichs and Zakzanis, 1998; San et al., 2007; Tuulio-Henriksson et al., 2011). Both neurocognitive deficits and limitations in the ability to carry out daily activities could contribute to poor circumstances in daily life, exaggerating negative attitudes, thus contributing to lower motivation, interest, and engagement in productive activities.

Neurocognitive dysfunction is also a key aspect of BD (Lewandowski et al., 2011), observable even during the remission of symptoms (Torres et al., 2007; Bora et al., 2011; Mann-Wrobel et al., 2011; Gama et al., 2013), with a strong impact on social functioning (Huxley and Baldessarini, 2007; Martino et al., 2009; Wingo et al., 2010). A meta-analysis by Kurtz and Gerraty (2009) considering 42 studies including euthymic BD patients (e.g., Bora et al., 2009a) stated that BD is characterized by an overall level of moderate cognitive impairment, that may exacerbate during acute phases, having a direct effect on rehabilitation outcome and an indirect effect on SC (Bell et al., 2009). Neuroimaging studies in SKZ have linked structural and functional abnormalities to symptoms and progressive structural changes to clinical course and functional outcome (Ahmed et al., 2013). Alterations in brain structures has been found also in BD, more pronounced in patients with repeated episodes (Gama et al., 2013).

Social cognition, defined as the mental operations underlying social interactions (Green et al., 2005), is considered a multidimensional domain, involving a complex set of processes allowing adaptive social interaction as the representation of internal somatic state, the awareness of the self-perception of others and interpersonal motivation (Fiske and Taylor, 1991; Kunda, 1999; Amodio and Frith, 2006). Both SKZ and BD patients show deficits in SC tasks, mainly in those requiring greater context sensitivity, performing normally in tasks that can be solved by explicit knowledge (Baez et al., 2013). Previously, Bromley and Brekke (2010), measuring social functioning in SKZ, highlighted how explicit knowledge is not enough to perform well in real life, identifying three dimensions of functioning: functional capacity, functional performance, and functional outcome. In particular functional capacity is the ability to perform a functional task (capacity) while functional performance is the ability to perform (performance) the same task in the community environment. Functional outcomes are the result of both capacity and performance; indeed, an individual may demonstrate a good functional capacity but may not be able to use it in his own social context. Recently, Pinkham et al. (2013) identified four core domains of SC: emotion processing, social perception, theory of mind/mental state attribution, and attributional style/bias. They focused on one particular aspect of SC, known as “Theory of Mind” (ToM) or “mentalizing” conceptualized as the ability to reflect upon one's own and other persons' mental states including desires, beliefs, knowledge, intentions, and feelings (Frith and Frith, 2003), repeatedly shown to be compromised in most SKZ patients (Lee et al., 2004) and evaluable with a variety of tasks and assessment methods (Brüne and Brüne-Cohrs, 2006). Most common tests utilized to assess ToM abilities are the Hinting Task (Corcoran et al., 1995), the cartoon method (Corcoran et al., 1997; Brüne, 2003), the pictorial tasks (Sarfati et al., 1997), ToM Advanced Test—composed of stories and drawings—created by Happé (1994), the “Moving Shapes” paradigm, used in early stages of SKZ (Abell et al., 2000; Koelkebeck et al., 2010), the Eyes Test designed to assess the capacity to re-attribute complex mental states in adults and adolescents in absence of severe mental retardation (Baron-Cohen et al., 2001; Serafin and Surian, 2004), the Faux Pas Test evaluating the ability to recognize a social faux pas (Baron-Cohen et al., 1999).

In SKZ neural mechanisms underlying metacognition, defined as the processes by which we monitor and control our own cognitive processes (Frith, 2012), include frontal lobe, in particular fronto-temporal and fronto-parietal circuits, premotor cortex, mirror neurons and dopaminergic reward circuits, involving neuropeptides such as oxytocin and vasopressin (Gallese and Goldman, 1998; Chafee and Goldman-Rakic, 2000; Mehta et al., 2013). Interestingly, most cortical abnormalities are subject to regional variations and differ from those observed in neurodegenerative diseases. Gray matter reductions in “social brain” areas of SKZ patients such as temporal and left occipital white matter regions, left posterior callosal region pole and left anterior hippocampus seem to be involved in socioemotional processing including ToM (Olson et al., 2007; Schobel et al., 2009; Miyata et al., 2010).

BD patients, both during mood phases and euthymic states, revealed impaired emotion processing with poor ability to distinguish facial emotions and impaired ToM (Bozikas et al., 2006; Summers et al., 2006; Lahera et al., 2008; Sánchez-Moreno et al., 2009; Montag et al., 2010; Martino et al., 2011). The processing of facial expressions of others relies upon the neural system of ventral prefrontal cortex (VPFC), amygdala and their interconnections, disrupted in BD patients (Blumberg et al., 2003; Lochhead et al., 2004; Adler et al., 2005; Stanfield et al., 2009; Gama et al., 2013; Lim et al., 2013).

Different brain regions seem to undergo different domains of SC: in some studies amygdala volume was correlated to impaired facial emotion recognition (FER) ability, whereas medial prefrontal cortex volume was correlated to impaired emotion attribution (Yamada et al., 2007; Matsukawa and Murai, 2013). Furthermore, ventral striatum, which is implicated in emotional and motivational aspects of behavior, seem to have an important function for SC ability (Adolphs, 2001). ToM studies in euthymic BD patients (Montag et al., 2010) revealed, independently from cognitive deficits, an insufficient performance in cognitive ToM with preserved emotional mentalizing abilities correlated with the number of manic episodes (Kerr et al., 2003; Olley et al., 2005; Lahera et al., 2008). A recent electrophysiological study by Ibañez et al. (2013) has found emotional N170 impairment in SKZ and BD patients, being cortical processing of emotional stimuli predictive of social-cognitive profile, indexed by measures of ToM, fluid intelligence, speed processing and executive functions. Previously, a comparison between euthymic BD patients and controls, pointed out abnormal facial modulation associated with individual profiles of ToM in BD patients (Ibañez et al., 2012).

In summary, neuropsychological and SC deficits are present both in SKZ and in BD, involving several brain areas, among which frontal lobes seem to play a crucial role.

Until now in literature neuropsychological findings have been mainly obtained with classical cognitive measures, however, more context-sensitive measures similar to real-life situations should be used when studying major psychoses (Baez et al., 2013). For this reason, in our study, we administered the Executive and Social Cognition Battery (ESCB), proved to be more sensitive in detecting executive and social cognitive impairments than conventional batteries, both in early behavioral variant of frontotemporal dementia (bvFTD) and BD (Torralva et al., 2009, 2012). Previous studies highlighted the importance of including ecological tests in the assessment of BD patients in order to provide a more realistic cognitive profile of this patient population, allowing better therapeutic and rehabilitation strategies able to minimize impact in real-life settings (Torralva et al., 2012).

## Aim of the study

The objective of the study was to analyze neurocognitive abilities, SC and global functioning in a pharmacologically stabilized sample of SKZ, BD patients in comparison to HC, using a specific neuropsychological and SC battery in an ecological context to analyze a possible correlation with subjects' global assessment of functioning.

## Materials and methods

### Sample

Forty-eight outpatients were enrolled at the Department of Psychiatry, Fondazione IRCCS Ca' Granda, Ospedale Maggiore Policlinico University of Milan: 30 stabilized SKZ patients (10 paranoid, 14 undifferentiated, 6 disorganized subtypes) and 18 euthymic BD patients (10 BD I, 8 BD II). Age-matched HC (*n* = 18) were recruited among volunteers who did not have a history of drug abuse. This study was approved by the Ethics Committee of Fondazione IRCCS Ca' Granda Maggiore Policlinico Hospital, Milan and informed consent was obtained from all subjects. Patients' inclusion criteria were a diagnosis of SKZ or BD according to Diagnostic and Statistical Manual for Mental Disorders-Text Revision (DSM-IV-TR). Exclusion criteria were: acute psychotic episodes in SKZ referring to Positive and Negative Symptom Scale (PANSS) (Kay et al., 1987) with a score >50; acute depression episodes in BD referring to Hamilton Depression Rating Scale scores (HDRS > 7) (Hamilton, 1960); acute mania episodes in BD referring to Young Mania Rating Scale scores (YMRS > 10) (Young et al., 1978); mental retardation or other neurological brain diseases.

### Evaluation tools

Trained psychiatrists conducted the Structured Clinical Interview for DSM Axis I (First et al., 2002) and rated patient functioning at baseline. The following psychometric scales were administered to SKZ patients: PANSS (Kay et al., 1987), Calgary Depression Scale for Schizophrenia (CDSS) (Addington et al., 1990) and Clinical Global Impression (severity of illness) (CGIs) (Guy, 1976). HDRS (Hamilton, 1960), YMRS (Young et al., 1978), and Hamilton Anxiety Scale (HAM-A) (Hamilton, 1959) were administered to BD patients. Global functioning (social, functional, and occupational) for both patients' groups and HC was measured with General Assessment of Functioning scale (GAF) included in DSM-IV-TR (American Psychiatric Association, 2000).

### Neurocognitive assessment

Cognitive status of both HC and patients (SKZ and BD) was assessed through standard neuropsychological battery: the Brief Assessment of Cognition in Schizophrenia (BACS) (Keefe et al., 2004; Anselmetti et al., 2008). In our study we applied a battery with normative data available for the Italian population.

Among cognitive batteries BACS (Keefe et al., 2004) can be easily administered and scored and it has been used in several SKZ clinical trials (Keefe et al., 2008). BACS assess different domains of cognitive function (Verbal Memory, Working Memory, Motor Speed, Attention, Verbal Fluency, and Executive Functions) with six tests, lasting about 35 min. Keefe et al. (2006) suggest that BACS scores are correlated with a performance-based measure of functional capacity and real-world functional outcome. It is noteworthy that BACS has also high test–retest reliability properties which are important for assessing alteration over time (Anselmetti et al., 2008).

Following is a description of the 6 subtests of the BACS:

– List Learning (Verbal Memory): Subjects are read a list of 15 words and then asked to recall as many as possible. This procedure is repeated five times and designed to measure episodic memory functions.
– Digit Sequencing Task (Working Memory): Subjects are presented with randomly ordered clusters of numbers with steadily increasing trial length. They are asked to report the numbers from lowest to highest.
– Token Motor Task (Motor Speed): Subjects are given 100 plastic tokens and asked to pick up one token with each hand simultaneously as rapidly as possible for 1 min and place them into a container.
– Verbal Fluency: (1) Semantic Fluency: Subjects are given 1 min to produce as many different words as possible within the animal category. (2) Letter Fluency: In two separate trials, subjects are given 1 min to produce as many words as possible that begin with a given letter, here T and R.
– Tower of London Test (Executive Functions/Reasoning and Problem Solving): Subjects look at two pictures simultaneously. Each shows three different-colored balls arranged on three pegs, with the balls in a unique arrangement in each picture. The person is required to accurately estimate the total number of times the balls in one picture would have to be moved in order to make the arrangement of balls identical to that of the other opposing picture, while employing the standard rules employed in tower tests (balls are moved one at a time and balls on top of other balls must be moved first).
– Symbol Coding (Attention and Processing speed): in this test, subjects write numerals 1–9 as matches to non-meaningful symbols on a response sheet for 90 s, as based on a key provided to them.

### Executive and social cognitive measures

All participants (both HC and patients) completed ecological tasks included in the ESCB (Torralva et al., 2009). This battery was created in order to detect cognitive and social components of the early stages of bvFTD, consisting of five subtests. Some tasks were used largely in neurological and neurorehabilitation fields (e.g., Manes et al., 2009; Torralva et al., 2009). Shallice and Burgess (1991) first demonstrated that patients with frontal lobe damage may be specifically impaired in everyday situations that require planning and multitasking, despite normal performance on standard cognitive tests. These authors found that three patients with frontal lobe syndrome due to traumatic brain damage performed well on a wide range of conventional executive tests, but showed noticeable difficulties with two novel tasks, where they had to organize their behavior and set priorities in the face of competing demands. The use of ecological tests could be helpful in psychiatric studies considering that patients have a large range of neuropsychological impairments.

Below is a description of the subtests of the ESCB.

The Hotel Task: “Hotel task” is part of a number of ecologically valid tests of executive function, in which individuals are required to carry out five hotel-related tasks, e.g., making up guests' bills, sorting coins, proofreading a brochure. Patients were required to devote some time to each test having only 15 min. Patients also have to keep in mind to press a button at two pre-designated times that correspond to opening and closing the hotel garage gate. Performance is measured in three ways, number of tasks attempted, deviation from optimal time on each task, and opening and closing the garage gate (Manly et al., 2002; Torralva et al., 2009).
MET-HV: As regards to Multiple Errands Test (MET) (Shallice and Burgess, 1991), a multitasking test carried out in shopping context, we adapted the 2002 test version (Knight et al., 2002) created for hospital settings (MET-HV). Its strengths are the simplicity of administration and the few administration time, being a good indicator of functional performance. The test requires carrying out a number of tasks simulating “real life” situations in which minor inconveniences can take place. The test takes place inside the hospital: the patient has a card with several sets of simple tasks with 12 subtasks. The first set requires participants to attain six specific goals, which include collecting an envelope from the secretary, purchasing three items (a postcard, a letter, a bottle of mineral water), using the internal phone and posting something to an external address. The second set involves obtaining and writing down on a chart some information (the price of a snack, how many parking spaces are available for visitors in the hospital, at what time the hospital's bar opens and closes on Friday and Saturday). The participant is required to call the examiner about 20 min after the test has begun and state the time over the phone. The third task requires the participant to inform the examiner when the task has been completed. Rules are clearly stated in the instruction sheet and the participant's behavior, while carrying the tasks, is monitored by two examiners. Errors in this test were categorized as: (a) inefficiencies, where a more effective strategy could have been applied; (b) rule breaks, where a specific rule was broken; (c) interpretation failure, where the requirements of a task had been misunderstood; (d) task failures and (e) total fails, the sum of all the previous ones.
MET is often utilized as an assessment tool useful for the detection of deficits in real-life executive functioning in post-stroke patients (Rand et al., 2009) and among patients with vascular lesions (Manes et al., 2009). Dawson et al. (2005b) showed a good correlation of this task with self-report measures of every day ability and living skills in cerebrovascular accident patients.
1. IOWA Test: This test represents a version of Becharaand colleagues test (1994), firstly used in neurological settings (patients with prefrontal cortex damage). This is a gambling task that models real life personal decision making activities in real time that include reward and punishment and the uncertainty of outcomes. The task involves four decks of cards, called A, B, C, and D. Subjects must choose one card at a time from one of the four decks. Desks A and B are ultimately risky (large rewards and large punishment) while C and D are more conservative (small rewards and small loss). The task is completed after 100 selections. Net scores are calculated with following formula: [(C + D) − (A + B)]; positive net scores reflect advantageous performance whereas negative net scores reflect the disadvantageous performance (Bechara et al., 2000). The IOWA Gambling Task (IGT) was putatively associated with ventromedial frontal lesions, and show decision-making deficits manifest in consistent selection of risky decks (Bechara et al., 2000; Torralva et al., 2007). The task is influenced by cognitive functions besides reward coding and use, including learning, shifting, and spatial working memory (Dunn et al., 2006).
2. Reading the Mind in the Eyes Test: We adopted Italian version of this ToM task (Baron-Cohen et al., 2001; Serafin and Surian, 2004): Participants are required to choose between four options (adjectives) that best describes what the person in the presented photo is thinking or feeling. Adjectives and descriptions used refer to complex states as embarrassment and shame. Complex emotions rise after understanding basic emotions and metarepresentative ability (Tangney and Fischer, 1995; Surian, 2002).
3. Faux Pas Test: We adopted the “Faux Pas Recognition Test” (adult version by Stone et al., 1998; Baron-Cohen et al., 1999). Participants are asked whether something inappropriate was said in some stories that they have to read and that may contain a social faux pas. In order to understand that a faux pas has occurred, the subject has to represent two mental states: first that the person committing the faux pas is unaware that he has said something inappropriate (cognitive theory of mind) and, second, that the person hearing it might feel hurt or insulted (affective theory of mind).

### Statistical analyses

Pairwise comparisons between groups were performed using χ^2^ test for categorical variables and two-sample Wilcoxon (Mann-Whitney) rank sum for quantitative variables. Multiple regression models with robust standard error were also used to compare GAF, neuropsychological, and ecological measures across groups while adjusting for gender, age, and past use for alcohol or drugs.

Spearman's rho correlation coefficient was used to quantify the relationship between GAF and neuropsychological or ecological measures. Sidak correction for multiple correlations was performed.

Significance level for all statistical tests was set at 0.05, two-tailed.

Statistical analyses were performed using Stata SE (StataCorp. 2011. Stata Statistical Software: Release 12. College Station, TX: StataCorp LP).

## Results

SKZ patients differ significantly from HC subjects (*p* = 0.001) and BD patients (*p* < 0.001) for substance abuse and gender, with a greater percentage of males, while no gender differences were found between BD patients and HC (*p* = 0.45). A more frequent history of substance abuse was found among SKZ patients with respect to the other two groups (*p* = 0.02). No significant differences were found between the groups for age, age at onset, duration of illness, and duration of untreated illness (Table 1).

**Table 1:** **Differences between healthy controls (HC), bipolar disorder (BD) and schizophrenia (SKZ) patients in demographic and clinical measures**.

**Variables**	**HC**	**BD**	**SKZ**
Total sample	*n* = 18	*n* = 18	*n* = 30
Gender	Males *n* = 6	Males *n* = 4	Males *n* = 24
P HC vs. BD = 0.45	Females *n* = 12	Females *n* = 14	Females *n* = 6
P HC vs. SKZ = 0.001			
P BD vs. SKZ < 0.0001			
Age (years)	36.11 ± 14.51	42.22 ± 11.72	42.47 ± 10.24
Age at onset (years)	–	24.72 ± 11.47	21.20 ± 3.90
Duration of illness (years)	–	17.50 ± 12.99	21.27 ± 11.59
DUI (years)	–	4.89 ± 7.40	3.23 ± 5.04
Global assessment of functioning	90.5 ± 7.03	67.39 ± 8.79	44.8 ± 10.87
Hamilton depression rating scale	3.39 ± 1.72	4.78 ± 2.69	–
Young mania rating scale	3.22 ± 1.96	2.55 ± 2.20	–
Hamilton anxiety scale	0.72 ± 0.83	4 ± 2.50	–
Positive and Negative Syndrome Scale	–	–	50 ± 6.44
Calgary depression scale for schizophrenia	–	–	4.93 ± 4.16
Previous abuse P BD vs. SKZ = 0.02	0/18	5/18 (Marijuana 2/5, Cocaine 0/5, Alcohol 3/5, Heroin 0/5)	19/30 (Marijuana 14/19, Cocaine 5/19, Alcohol 12/19, Heroin 1/19)
Medications		18/18	30/30
		(Atypical antipsychotic 16/18, Typical antipsychotic 3/18, Mood stabilizer 18/18, Antidepressant 8/18, Benzodiazepine 5/18)	(Atypical antipsychotic 19/30, Typical antipsychotic 11/30, Mood stabilizer 4/30, Antidepressant 2/30, Benzodiazepine 8/30)

DUI = Duration of Untreated Illness.

Statistical analysis adjusted for abuse, age, and gender confirmed that our results were not influenced by these variables (Tables 2, 3).

**Table 2:** **Performances of healthy controls (HC), bipolar disorder (BD) and schizophrenia (SKZ) patients on brief assessment of cognition in schizophrenia (BACS)**.

**BACS subtests**	**HC (*n* = 18)**	**BD (*n* = 18)**	**SKZ (*n* = 30)**	**HC vs. BD**	**HC vs. SKZ**	**BD vs. SKZ**
List learning (verbal memory)	54.9 ± 9.7	47.1 ± 9.9	37.4 ± 7.8	*P-c* = 0.01	*P-c* < 0.0001	*P-c* = 0.004
	(n.v. = 38.36)	(n.v. = 38.36)	(n.v. = 38.36)	*P-a* = 0.04	*P-a* < 0.0001	*P-a* = 0.008
Digit sequencing task (working memory)	24.3 ± 2.8	20.3 ± 5.2	17.6 ± 5.1	*P-c* = 0.02	*P-c* < 0.0001	*P-c* = 0.08
	(n.v. = 17.66)	(n.v. = 17.66)	(n.v. = 17.66)	*P-a* = 0.002	*P-a* < 0.0001	*P-a* = 0.03
Token motor task	79.7 ± 10.4	69.8 ± 15.2	62.6 ± 14.9	*P-c* = 0.04	*P-c* = 0.0002	*P-c* = 0.12
	(n.v. = 76.03)	(n.v. = 76.03)	(n.v. = 76.03)	*P-a* = 0.02	*P-a* < 0.0001	*P-a* = 0.05
Symbol coding	61.0 ± 12.2	48.2 ± 8.6	39.5 ± 11.2	*P-c* = 0.002	*P-c* < 0.0001	*P-c* = 0.004
	(n.v. = 46.35)	(n.v. = 46.35)	(n.v. = 46.35)	*P-a* = 0.001	*P-a* < 0.0001	*P-a* = 0.005
Verbal fluency	55.3 ± 10.5	39.4 ± 11.0	35.5 ± 9.0	*P-c* = 0.0004	*P-c* < 0.0001	*P-c* = 0.34
	(n.v. = 39.18)	(n.v. = 39.18)	(n.v. = 39.18)	*P-a* < 0.0001	*P-a* < 0.0001	*P-a* = 0.23
Tower of London	16.6 ± 2.7	14.5 ± 3.8	13.4 ± 3.4	*P-c* = 0.08	*P-c* = 0.002	*P-c* = 0.23
	(n.v. = 14.24)	(n.v. = 14.24)	(n.v. = 14.24)	*P-a* = 0.02	*P-a* < 0.0001	*P-a* = 0.06

P-c = P-crude; P-a = P-adjusted (Multiple regression model—for abuse).

Values are shown as Mean (SD).

n.v. = normal value, referred to an equivalent score 2 (Anselmetti et al., 2008).

**Table 3:** **Performances of healthy controls (HC), bipolar disorder (BD) and schizophrenia (SKZ) patients on executive and social cognition battery (ESCB)**.

**ESCB subtests**	**HC (*n* = 18)**	**BD (*n* = 18)**	**SKZ (*n* = 30)**	**HC vs. BD**	**HC vs. SKZ**	**BD vs. SKZ**
MET-HV task attempted	12.0 ± 0.0	10.6 ± 1.5	10.1 ± 2.3	*P-c* = 0.0003	*P-c* = 0.0001	*P-c* = 0.59
				*P-a* = 0.007	*P-a* = 0.002	*P-a* = 0.26
MET-HV task failures	0.0 ± 0.0	1.4 ± 1.5	1.9 ± 2.3	*P-c* = 0.0003	*P-c* = 0.0001	*P-c* = 0.59
	(0.4 + 0.2^*^)	(0.4 + 0.2^*^)	(0.4 + 0.2^*^)	*P-a* = 0.007	*P-a* = 0.002	*P-a* = 0.26
MET-HV inefficiences	0.3 ± 0.5	1.1 ± 1.0	1.5 ± 0.8	*P-c* = 0.008	*P-c* <0.0001	*P-c* = 0.08
	(0.06 + 0.4^*^)	(0.06 + 0.4^*^)	(0.06 + 0.4^*^)	*P-a* = 0.009	*P-a* = 0.002	*P-a* = 0.29
MET-HV rules breaks	0.0 ± 0.0	0.9 ± 0.5	0.6 ± 0.6	*P-c* < 0.0001	*P-c* = 0.0001	*P-c* = 0.07
	(0.63 + 0.4^*^)	(0.63 + 0.4^*^)	(0.63 + 0.4^*^)	*P-a* < 0.0001	*P-a* = 0.004	*P-a* = 0.29
MET-HV interpretation failures	0.0 ± 0.0	0.2 ± 0.4	0.6 ± 1.0	*P-c* = 0.04	*P-c* = 0.002	*P-c* = 0.15
	(0.24 + 0.2^*^)	(0.24 + 0.2^*^)	(0.24 + 0.2^*^)	*P-a* = 0.05	*P-a* = 0.002	*P-a* = 0.01
Hotel task task attempted	5.0 ± 0.0	4.7 ± 0.6	3.2 ± 1.3	*P-c* = 0.04	*P-c* < 0.0001	*P-c* < 0.0001
	(4.9 + 0.1^*^)	(4.9 + 0.1^*^)	(4.9 + 0.1^*^)	*P-a* = 0.08	*P-a* < 0.0001	*P-a* < 0.0001
Hotel task time deviations (s)	2.1 ± 6.0	52.1 ± 98.3	266.4 ± 203.7	*P-c* = 0.04	*P-c* < 0.0001	*P-c* < 0.0002
	(319.90 + 42.9^*^)	(319.90 + 42.9^*^)	(319.90 + 42.9^*^)	*P-a* = 0.16	*P-a* < 0.0001	*P-a* < 0.0001
IOWA gambling task	12.0 ± 12.5	8.9 ± 22.6	−2.1 ± 21.4	*P-c* = 0.63	*P-c* = 0.006	*P-c* = 0.09
	(adv > 0^**^)	(adv > 0^**^)	(adv > 0^**^)	*P-a* = 0.44	*P-a* = 0.001	*P-a* = 0.02
The mind in the eyes test	25.7 ± 3.7	25.4 ± 3.6	19.6 ± 4.6	*P-c* = 0.79	*P-c* < 0.0001	*P-c* = 0.0001
	(23.1 + 5.1^***^)	(23.1 + 5.1^***^)	(23.1 + 5.1^***^)	*P-a* = 0.53	*P-a* < 0.0001	*P-a* < 0.0001
Faux pas test	18.1 ± 2.6	17.2 ± 2.3	12.7 ± 3.7	*P-c* = 0.12	*P-c* < 0.0001	*P-c* < 0.0001
	(19 + 1.5)	(19 + 1.5)	(19 + 1.5)	*P-a* = 0.29	*P-a* < 0.0001	*P-a* < 0.0001

P-c = P-crude; P-a = P-adjusted (Multiple regression model - for abuse, age, gender).

MET-HV = Multiple Errands Test-hospital version;

Values are shown as Mean (SD).

* Healthy controls values reported in Torralva et al.. study (2012).

** Advantageous net scores as calculated in Bechara et al. (2000).

*** Mean value in 41–60 age population as reported in “Test degli Occhi” (Serafin and Surian, 2004).

**Figure 1:**
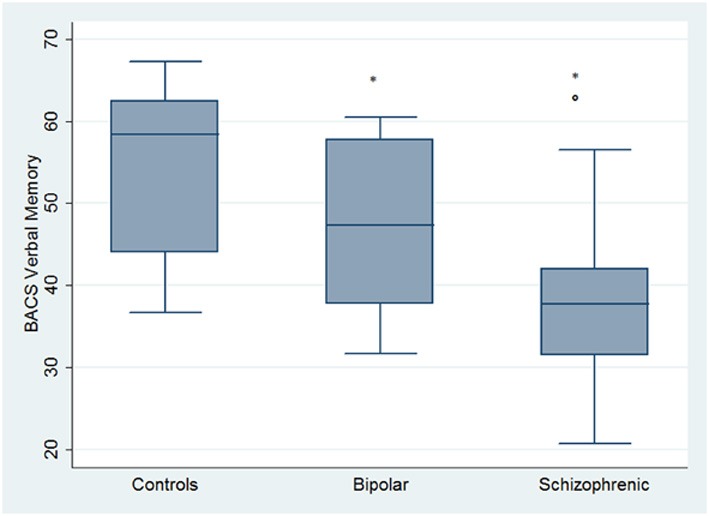
**Differences between groups in neuropsychological tasks: BACS verbal memory**. ^*^Significantly different from HC (*p* < 0.05). °Significantly different from BD (*p* < 0.05).

**Figure 2:**
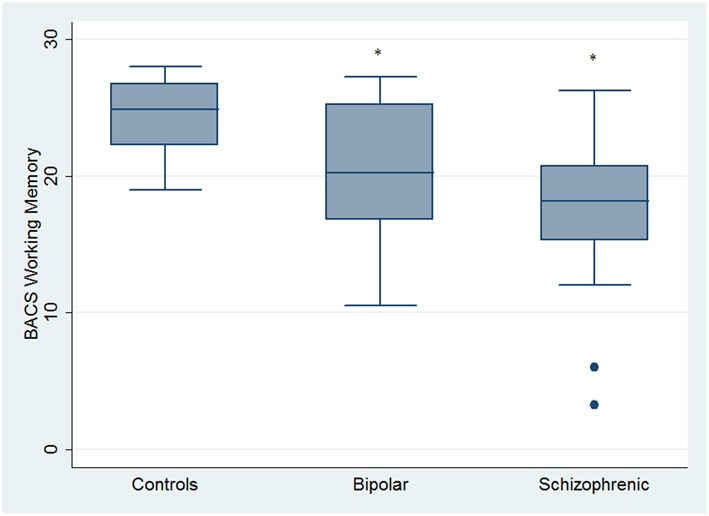
**Differences between groups in neuropsychological tasks: BACS working memory**. ^•^Outliers. ^*^Significantly different from HC (*p* < 0.05).

**Figure 3:**
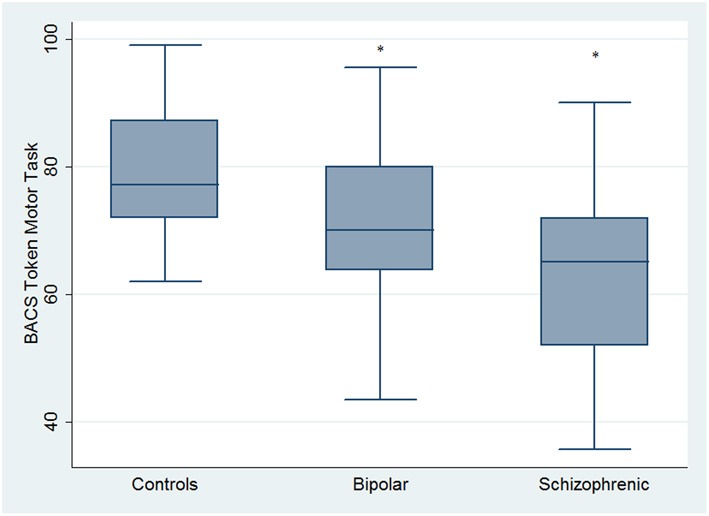
**Differences between groups in neuropsychological tasks: BACS motor speed**. ^*^Significantly different from HC (*p* < 0.05).

**Figure 4:**
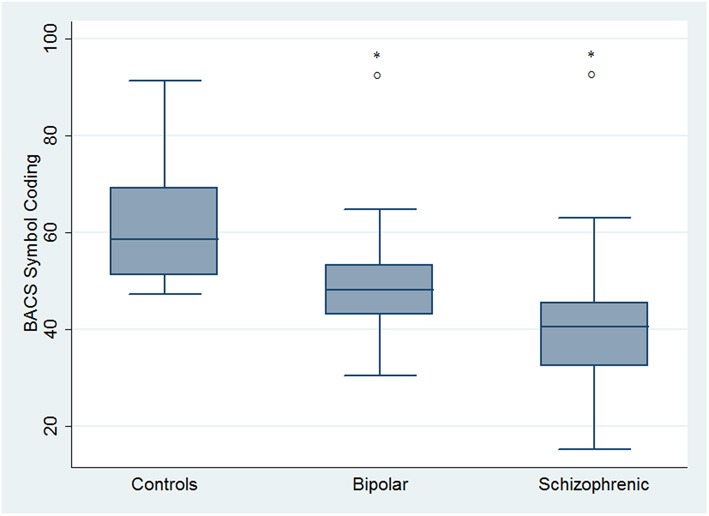
**Differences between groups in neuropsychological tasks: BACS symbol coding**. ^*^Significantly different from HC (*p* < 0.05). °Significantly different from BD (*p* < 0.05).

**Figure 5:**
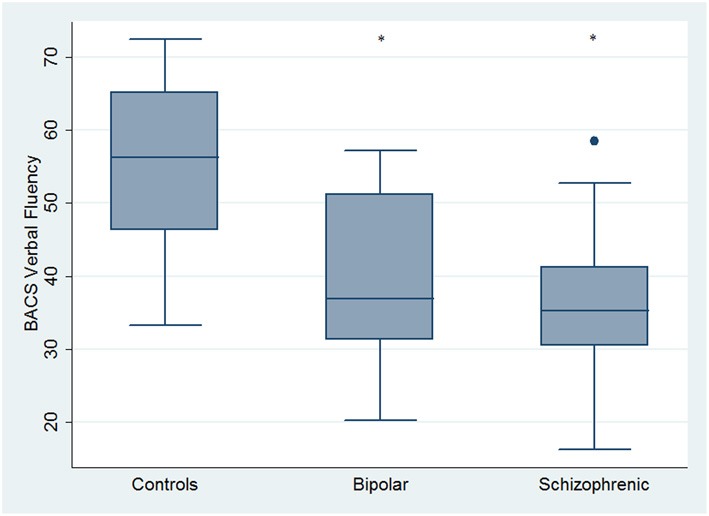
**Differences between groups in neuropsychological tasks: BACS verbal fluency**. ^•^Outliers. ^*^Significantly different from HC (*p* < 0.05).

Regarding neuropsychological tasks both SKZ and BD subjects showed in BACS test a significant worse performance compared to HC in verbal memory (for BD: *z* = 2.437; *p* = 0.01; for SKZ: *z* = 4.782; *p* < 0.001), working memory (for BD: *z* = 2.279; *p* = 0.02; for SKZ: *z* = 4.496; *p* < 0.001), motor speed (for BD: *z* = 2.010; *p* = 0.04; for SKZ: *z* = 3.749; *p* = 0.0002), symbol coding (for BD: *z* = 3.102; *p* = 0.002; for SKZ: *z* = 4.898; *p* < 0.001) and verbal fluency (for BD: *z* = 3.546; *p* = 0.0004; for SKZ: *z* = 4.835; *p* < 0.001) (Figures 1–5).

**Figure 6:**
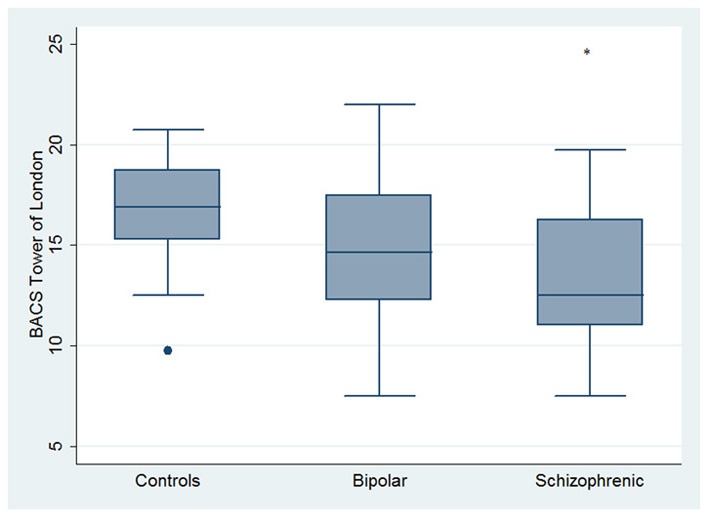
**Differences between HC and SKZ in neuropsychological tasks: BACS Tower of London test**. ^•^Outliers. ^*^Significantly different from HC (*p* < 0.05).

Performances differed significantly between BD and SKZ in BACS verbal memory (*z* = −2.897; *p* = 0.0038) and symbol coding (*z* = −2.854; *p* = 0.0043) (Figures 1, 4). Only SKZ patients obtained significantly lower scores compared to HC in Tower of London test (*z* = 3.079; *p* = 0.0021) (Figure 6).

**Figure 7:**
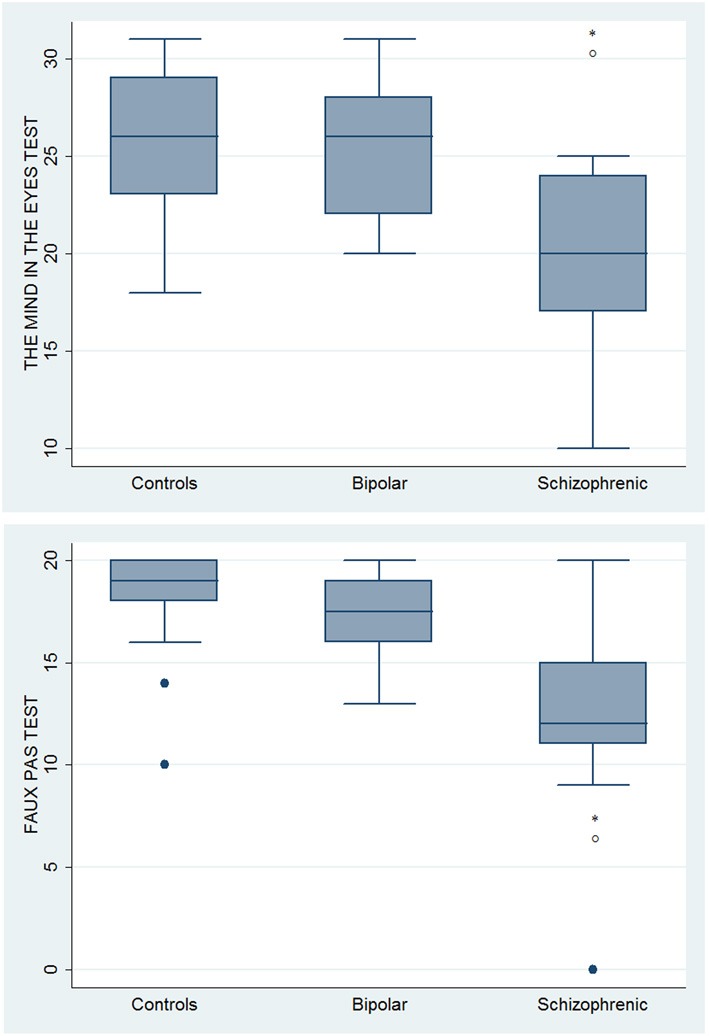
**Differences between groups in ESCB tasks: ToM tests**. ^•^Outliers. ^*^Significantly different from HC (*p* < 0.05). °Significantly different from BD (*p* < 0.05).

**Figure 8:**
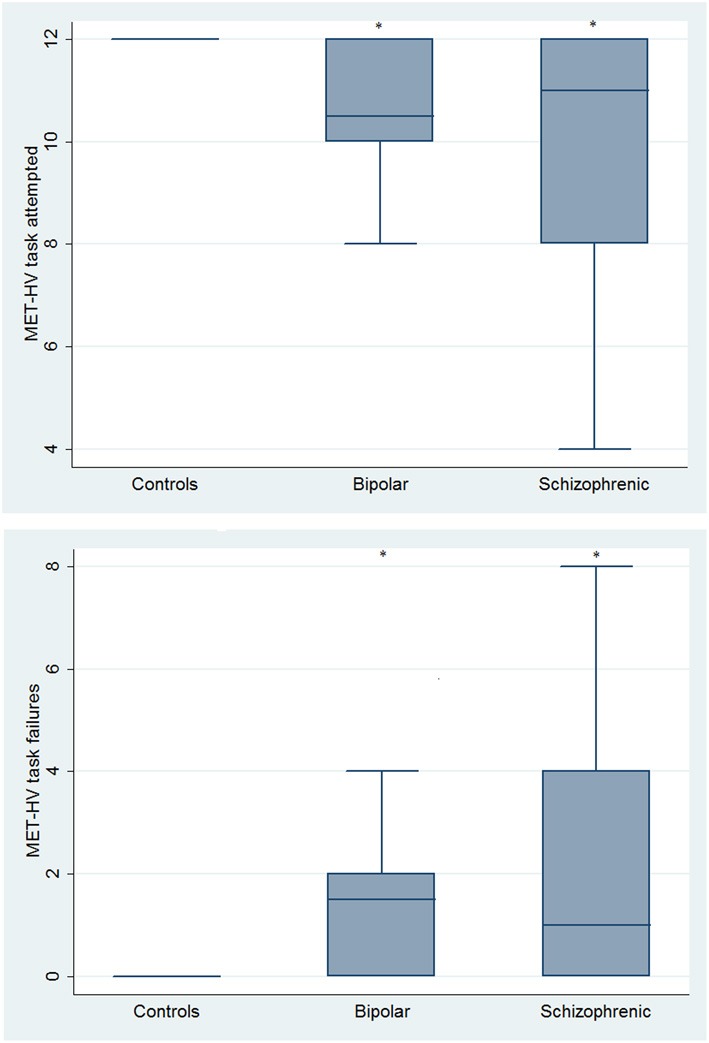
**Differences between groups in ESCB tasks: MET-HV Task Attempted and Task Failures**. ^*^Significantly different from HC (*p* < 0.05).

**Figure 9:**
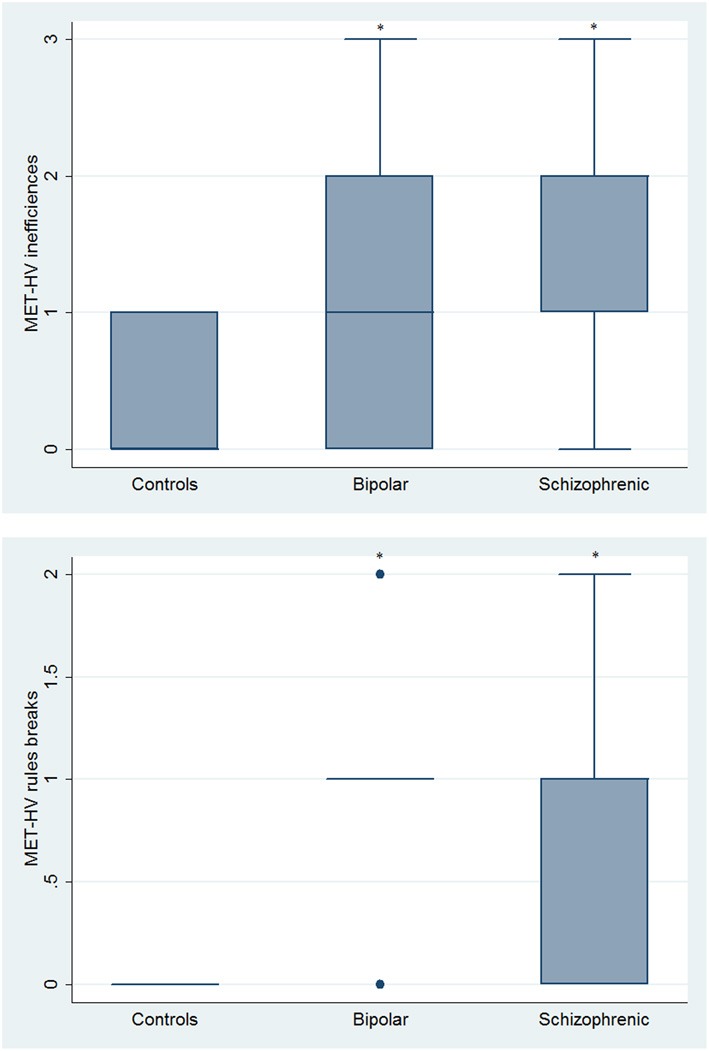
**Differences between groups in ESCB tasks: MET-HV Inefficiencies and Rule Breaks**. ^•^Outliers. ^*^Significantly different from HC (*p* < 0.05).

**Figure 10:**
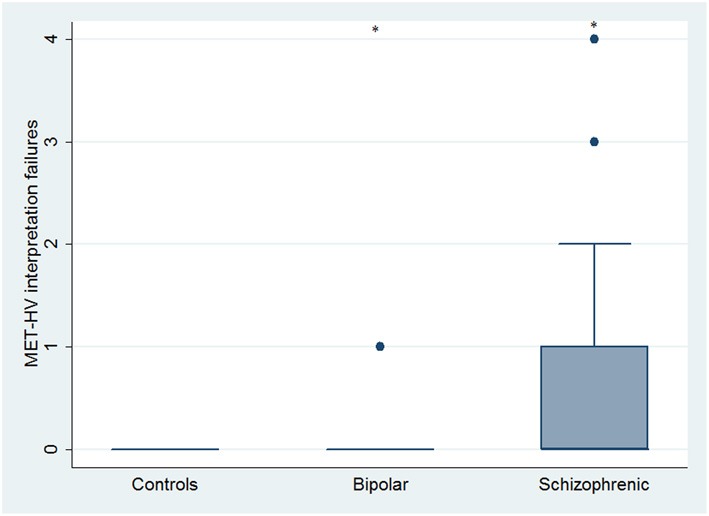
**Differences between groups in ESCB tasks: MET-HV Interpretation failures**. ^•^Outliers. ^*^Significantly different from HC (*p* < 0.05).

**Figure 11:**
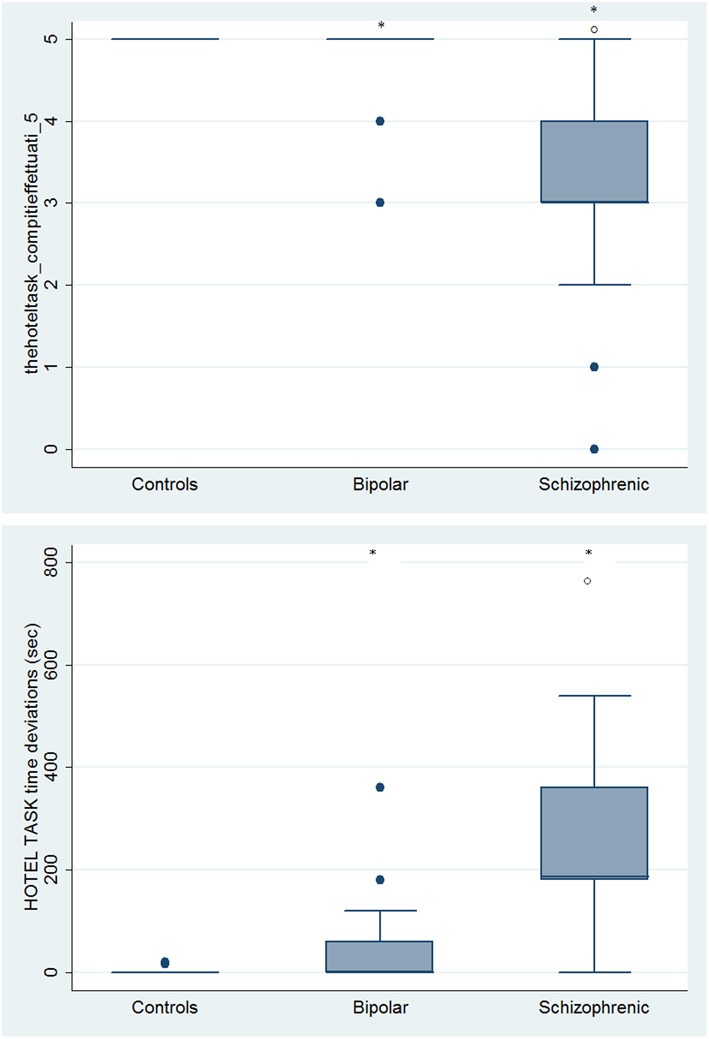
**Differences between groups in ESCB tasks: HOTEL Task Attempted and Time deviations**. ^•^Outliers. ^*^Significantly different from HC (*p* < 0.05). °Significantly different from BD (*p* < 0.05).

**Figure 12:**
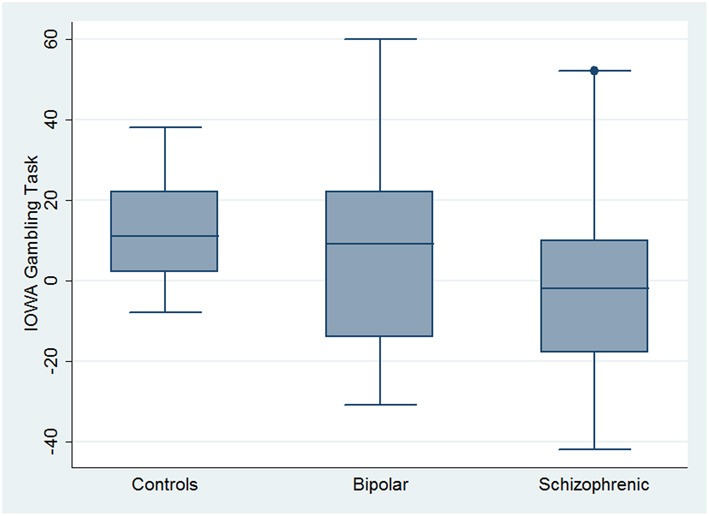
**Differences between groups in ESCB tasks: IGT**. ^•^Outliers.

With respect to SC tasks including ToM tests, SKZ patients performed worse than HC and BD in Eyes Test (*z* = 4.096, *p* < 0.001; *z* = −3.947; *p* = 0.001, respectively) and in Faux Pas Test (*z* = 4.410; *p* < 0.001; *z* = −4.138; *p* < 0.001, respectively), while BD patients performed similarly to HC (Figure 7). In MET-HV test, included in ESCB, both BD and SKZ subjects attempted to perform less tasks (for BD: *z* = 3.614, *p* = 0.0003; for SKZ: *z* = 3.819, *p* = 0.0001) (Figure 8), failed to complete a greater number of tasks (for BD: *z* = −3.614, *p* = 0.0003; for SKZ: *z* = −3.819, *p* = 0.0001) (Figure 8), broke more rules (for BD: *z* = −4.916, *p* < 0.001; for SKZ: *z* = −3.893, *p* = 0.0001) (Figure 9) and showed more interpretation failures (for BD: *z* = −2.092, *p* = 0.0365; for SKZ: *z* = −3.037, *p* = 0.0024) compared to HC (Figure 10). Moreover, both BD and SKZ subjects committed more inefficiencies (for SKZ: *z* = −4.538, *p* < 0.001; for BD: *z* = −2.645, *p* = 0.0082) than HC (Figure 9). In the Hotel Task, both patients' groups attempted to complete significantly less tasks (for BD: *z* = 2.089, *p* = 0.0367; for SKZ: *z* = 5.241, *p* < 0.01) and obtained greater time deviations in all tasks (for BD: *z* = −2.081, *p* = 0.0375; for SKZ: *z* = −4.750, *p* < 0.001) compared to HC (Figure 11). When comparing SKZ and BD subjects, SKZ patients showed less tasks attempted (*z* = −4.300, *p* < 0.001) and a greater sum of time deviation (*z* = 3.760 and *p* = 0.0002) (Figure 11). Finally, SKZ subjects' performed worse than HC in IGT (*z* = 2.740, *p* = 0.0061), while we didn't find any significant difference neither between BD and HC nor between SKZ and BD patients (Figure 12).

**Figure 13:**
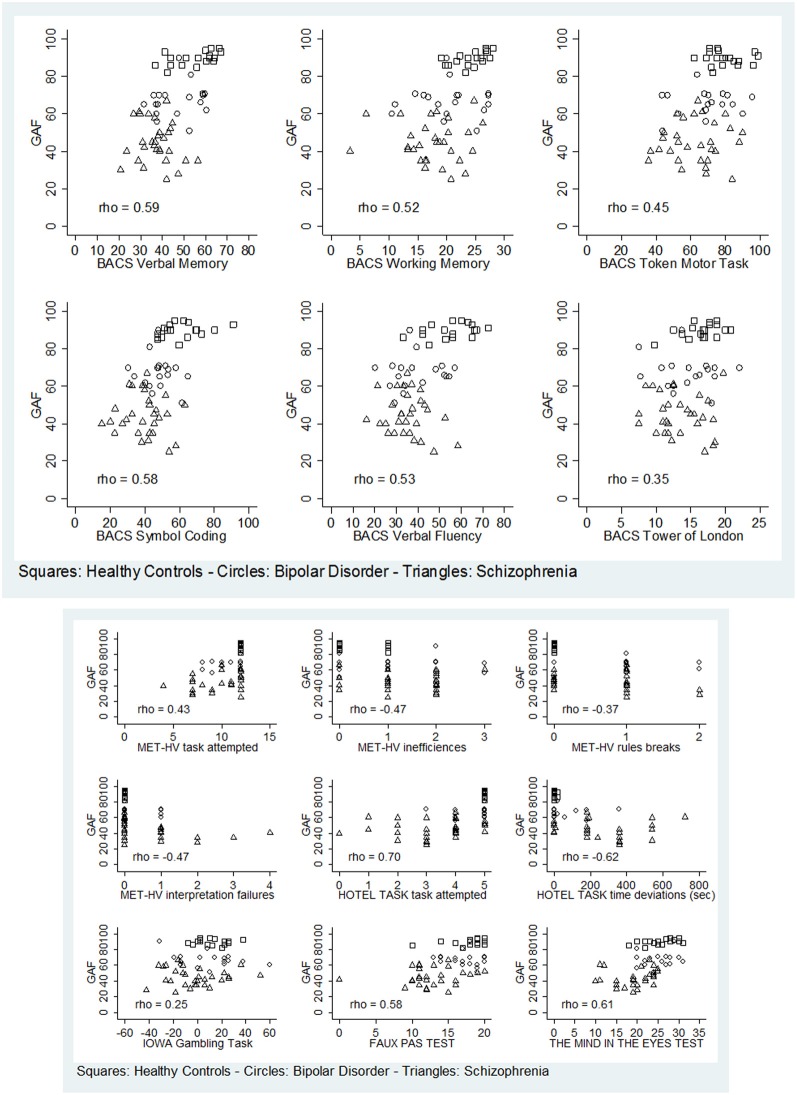
**Significant correlations between global functioning (GAF) and neuropsychological tasks (BACS)/ESCB tasks**.

Regarding global functioning, both BD and SKZ patients differed significantly from HC for GAF scores being inferior for patients (BD: *z* = 4.843, *p* < 0.001; SKZ: *z* = 5.758, *p* < 0.001). A significant difference was found also between SKZ and BD, with greater GAF scores for BD subjects (*z* = −5.204, *p* < 0.001). GAF scores were positively correlated to the performance in all BACS tests, except for Tower of London (verbal memory: rho = 0.5866, *p* < 0.001; working memory: rho = 0.5225, *p* = 0.0001; motor speed: rho = 0.4461, *p* = 0.0036; symbol coding: rho = 0.5799, *p* < 0.001; verbal fluency: rho = 0.5250, *p* = 0.0001), in both ToM tasks (Eyes test: rho = 0.6066, *p* < 0.001; Faux Pas Test: rho = 0.5847, *p* < 0.001) and to number of tasks attempted in MET-HV (rho = 0.4347, *p* = 0.0181) and Hotel Task (rho = 0.7024, *p* < 0.001) (Figure 13). Instead, GAF scores were negatively correlated to the number of task failures (rho = −0.4347, *p* = 0.0181), inefficiencies (rho = −0.4655, *p* = 0.0058) and interpretation failures (rho = −0.4653, *p* = 0.0059) in MET-HV and to the sum of time deviations in Hotel Task (rho = −0.6174, *p* < 0.001) (Figure 13).

On the contrary, performance in Tower of London (rho = 0.3473, *p* = 0.0861), rule breaking in MET-HV task and IGT performance did not seem to predict global functioning (rho = −0.3656 and 0.2473, *p* = 0.1511 and 0.9364, respectively) (Figure 13).

## Discussion

Among neuropsychological tests, a significant impairment was found both in SKZ and BD patients with respect to HC, with SKZ performances being worse than BD in the majority of tests. Data regarding impairment in verbal memory and executive functions were previously underlined by Altshuler et al. (2004) in both diseases. Verbal memory deficits, more pronounced in SKZ patients than in BD patients, have also been reported by Konstantakopoulos et al. (2011). Symbol Coding has been recently considered the most sensitive test in detecting attention and speed processes, seen as the early signs of deterioration in a variety of neurological disorders (Strauss and Brandt, 1986; Storandt and Hill, 1989; Lezak, 1995; Wechsler, 2008).

Interestingly our work shows differences among SKZ, BD, and HC performances in ecological tests, able to detect more subtle cognitive deficits, indicating that mainly SKZ but also BD patients could have a worse capacity for planning, low flexibility, and organization skills as previously reported by Torralva et al. (2009). Our findings are in line with some previous literature results, showing a worse performance for SKZ than for BD subjects both in standard cognitive domains, ToM tasks and experimental tasks (mainly in MET-HV and Hotel task) (Sanchez-Morla et al., 2009). It is important to note that our sample of SKZ patients present a significant disability in detecting both emotional (Eyes Test) and verbal (Faux Pas Test) ToM tasks as an evidence of global compromised ToM ability. In particular, in the Eyes Test SKZ patients are more compromised than BD patients in mental state decoding of other individuals' facial expressions, in contradiction to Donohoe et al. (2012), who state comparable levels of impairment in the two disorders. Furthermore, our findings showed a deficit in the Faux Pas Test in both groups, being SKZ patients more compromised; in line with previous studies reporting serious ToM deficits, both in BD and SKZ, demonstrated by poor performance in advanced ToM tasks, such as the recognition of a faux pas, a social misstep (Bertrand et al., 2007; Brüne et al., 2007; Bora et al., 2009a).

Failures in cognitive ToM (Faux Pas Test) were observed in a previous study by Ibañez et al. (2012) also among BD patients when compared to HC; in that test patients' performance was significantly reduced, whereas the Reading The Mind in the Eyes test, measuring mainly affective ToM, showed a non-significant difference. Cognitive ToM refers specifically to the ability to infer the mental beliefs and states of others, while “affective” ToM (emotional) refers to the ability to infer the emotions of others (Barrera et al., 2013).

In IOWA test the SKZ group showed a poor ability in reasoning before acting and insensitivity to future consequences with respect to BD patients, although statistical significance was not reached. In addition, a significant difference between SKZ and BD patients was found in GAF functionality, in part possibly explained by deficits reported in previous tests. According to our results, all the tasks, especially ecological tests, were significantly correlated with GAF scores, being a possible useful marker of social functioning in major psychoses. Although our sample is composed of stabilized patients, we have to take into account that sub-syndromal mood changes, mainly depressive, could alter the mechanisms of social understanding, thus, worsening the ability to detect faux pas or embarrassing social situations and recognize basic and complex emotions.

Numerous studies have reported that “real-world” situations, reproduced in ecological tests (e.g., MET-HV), assess everyday life ability better than traditional tests and could be more prognostic (Burgess et al., 1998, 2006; Wilson et al., 1998; Knight et al., 2002; Alderman et al., 2003; Dawson et al., 2005a,b). Studies involving individuals with bvFTD have underlined how patients can score normally on neuropsychological test and reveals no abnormalities in brain imaging, but demonstrate notable defects in social interactions therefore it is necessary to consider the context as an intrinsic part of SC. The social context network model (SCNM) has been linked to a fronto-insular-temporal circuit and seems to be involved in SC, attempting to update context, coordinate internal and external processes and associate previous information (Ibañez and Manes, 2012). Given the overlap of certain symptomatic dimensions between bvFTD and psychiatric disorders (apathy, disinhibition, depression, anhedonia, stereotyped behavior, and psychosis), such as late onset SKZ and BD (Pose et al., 2013), it could be hypothesized that the abnormal social context processing could explain SC deficits also in major psychoses.

We identified some problem areas in our study. Our sample, SKZ and BD patients differed significantly for substance (mainly cocaine, cannabis), alcohol abuse and gender. Alcohol and substance abuse have often proved to have an influence on cognitive performance, particularly on immediate verbal learning, processing speed and working memory (Meijer et al., 2012). Despite the high prevalence of substance abuse (particularly cannabis) both in SKZ and BD disorders, study results are still inconclusive regarding the repercussions on neurocognitive functions (Coulston et al., 2007). Moreover, in our sample abuse occurred many years before the assessment and was substantially moderate. Regarding gender, our sample was mainly composed by males and a study by Bora et al. (2009b) indicated that female patients performed better in ToM tests. However, statistical analysis performed in our sample, proved that gender and abuse did not influence the performances. Furthermore, we should take into account that the current observed relations between SC, neurocognition, and clinical assessment, have been studied cross-sectionally and may not necessarily represent the longitudinal outcome. The fact that all patients were medicated, constitutes an additional limitation of the study, given that patients received different classes of drugs to modulate neurotransmitters, which are known to affect specific aspects of ToM (Montag et al., 2008). Our sample did not present significant comorbidities, except for the presence of some personality traits although without the configuration of a personality disorder. Finally, a limitation of our study could be the diagnostic variability. It is accepted in literature that outcome of SKZ patients is worse than BD, but partially similar to BD type 1 patients (Lewandowski et al., 2011), suggesting the presence of a continuum between a typically psychotic disorder such as SKZ and affective disorders with important psychotic features such as BD1. In particular, among SKZ, 10 had a diagnosis of paranoid SKZ, 14 of undifferentiated SKZ and 6 of a disorganized subtype. It has been demonstrated that there is a difference in outcome between paranoid and non-paranoid SKZ (Kendler et al., 1984) and that patients with undifferentiated SKZ showed less clinical and cognitive recovery than the others (Seltzer et al., 1997). More recently, Salokangas et al. (2002) argued that the disorganized subtype presented poorer outcome and low quality of life, although data in literature are still inconclusive. However, in the last years, the multi-factorial etiology and the evidence of a continuum between the disorder and the general population (Kaiser et al., 2011) identified an ultra-risk population, supporting a dimensional approach. In light of these considerations and of the substantial homogeneity of the three groups, the diagnostic differences within our sample had more than likely not influenced our results.

## Conclusion

Our work substantially confirms data presented in literature of a more severe impairment of SKZ than BD in cognitive and SC tasks. To our knowledge, this is the first attempt to compare BD, SKZ patients and HC with the application of tools derived from neurological context considering SC as a mediator more closely related to community functioning than neurocognition and a target for psychosocial and pharmacological interventions. The originality of our work consists in a more specific and in-depth assessment of SKZ functioning in comparison to BD and HC.

The future goal will be to confirm these data in a larger sample, study bipolar subtypes in order to see if BD patients type I have a similar social cognitive functioning to SKZ as previously reported (Lewandowski et al., 2011) and to establish if specific cognitive remediation tasks can have an impact on outcome as suggested by Ryan et al. (2013). Furthermore, it would be of interest to study high risk populations for psychoses (HR) as in Whitney's study 2013, which reported in youths at high risk of BD a significant impairment in social reciprocity possibly due to innate differences in brain development governing socio-emotional functioning or to disruptions in normal development caused by mood regulation difficulties. The assessment of SC, besides traditional neuropsychological tests, could provide new insight into major psychoses, perhaps contributing to understanding the neural basis of these disorders, considering that human brain is influenced by emotions and social stimuli.

### Conflict of interest statement

The authors declare that the research was conducted in the absence of any commercial or financial relationships that could be construed as a potential conflict of interest.
